# Learning from the COVID-19 pandemic to combat climate change: comparing drivers of individual action in global crises

**DOI:** 10.1007/s13412-021-00727-9

**Published:** 2021-12-06

**Authors:** Marijn H. C. Meijers, Christin Scholz, Ragnheiður “Heather” Torfadóttir, Anke Wonneberger, Marko Markov

**Affiliations:** 1 Amsterdam School of Communication Research, Department of Communication Science, University of Amsterdam, PO BOX 15791, 1001 NG Amsterdam, The Netherlands; 2 Department of Communication Science, University of Amsterdam, PO BOX 15791, 1001 NG Amsterdam, The Netherlands

**Keywords:** Climate change, Behavior change, COVID-19, Pro-environmental behavior

## Abstract

The COVID-19 pandemic and climate change are two global crises that require collective action. Yet, the inertia typically associated with behavior change to limit climate change stands in contrast to the speed associated with behavior change to stop the spread of COVID-19. Identifying the roots of these differences can help us stimulate climate-friendly behaviors. We assessed the extent to which a number of theory-based drivers underlie behaviors aiming to counter COVID-19 and climate change with an online survey (*N* = 534). We focused on the role of a number of drivers derived from prominent behavior change theories and meta-analyses in the field, namely, personal threat, threat to close others, threat to vulnerable others, fear, participative efficacy, injunctive and descriptive social norms, and governmental policy perceptions. We investigated (1) what drivers people perceived as most important to engage in behaviors that limit the spread of the COVID-19 pandemic and climate change and (2) the strength of the associations between these drivers and engaging in behaviors that limit the spread of the pandemic and climate change. Results highlight three key drivers for climate change action: *changing* perceptions of governmental policy and perceptions of threat to close others and *priming* participative efficacy beliefs.

COVID-19 has changed our world rapidly. Individuals, organizations, and governments have shown willingness and capacity to make profound changes to public life, surprisingly quickly (Cova 2020; Johns Hopkins University & Medicine, 2020). This stands in contrast to the inertia that is typically exhibited in the response to climate change (Munck af Rosenschöld et al. 2014; Whitmarsh et al. 2013). This difference in the speed of reactions to climate change and COVID-19 has surprised scientists (Galbraith & Otto 2020) and journalists alike (Segalov 2020). Both the COVID-19 pandemic and climate change constitute global crises that require collective action. Given these similarities, what drives individuals to act swiftly and drastically to address one crisis but not the other? We argue that similarities of the COVID-19 pandemic to the climate change crisis present a unique opportunity to draw lessons from this intense moment of public involvement, which can be utilized to stimulate climate-friendly behavior (Galbraith & Otto 2020; Schmidt 2021). To this end, we explore differences and similarities in key drivers of actions in response to COVID-19 and climate change. Stimulating key drivers of swift COVID-19 responses in the context of climate change may be a vital first step to counter climate change.

Based on prominent theories of behavior change (Hornik & Woolf 1999), we argue that (1) drivers which are central to COVID-19, but not (yet) to climate change action, and (2) drivers that are already important in both crises are promising levers for future interventions. Based on the reasoning put forward by Fishbein and Cappella (2006), the former are candidates for *change*: Introducing and fostering novel drivers in the climate change context that are known to be associated with rapid behavior change during the COVID-19 crisis may help also increase climate change action readiness. The latter, successful COVID-19 drivers that are already present in the climate change context, are candidates for *priming*: Emphasizing and reinvigorating existing drivers in the climate change context that are known to play a role in the rapid COVID-19 response can selectively leverage those bases of climate change behavior that are already present in the target group.

## Drivers of behavioral change and collective action

We evaluate these two criteria for a set of behavioral drivers from well-established behavioral change models, collective action theories, extensive previous research, and meta-analyses on behavioral change (Bergquist et al. 2019; Rainear & Christensen 2017; Rogers 1975; Urbanovich & Bevan 2020; van Valkengoed & Steg 2019; Witte & Allen 2000). Specifically, the Extended Parallel Process Model (Witte 1992) highlights the importance of perceived *threat* and *efficacy*. Furthermore, recent meta-analyses regarding collective action emphasize the central role of *social norms* (Bergquist et al. 2019; van Valkengoed & Steg 2019). Finally, we examine perceptions of *governmental policy*, following insights from the Behavior Change Wheel (Michie et al. 2011) and several scholars arguing that governmental policy plays a significant role in large-scale issues like pandemics and climate change (Cooper & Nagel 2021; Jagers et al. 2020).

### Threat and efficacy

Behavior change models like the Extended Parallel Process Model (Witte 1992) and Protection Motivation Theory (Rogers 1975) have proven valuable in predicting behaviors in response to threats, like diseases or climate change (Hartmann et al. 2014; Homburg & Stolberg 2006; Witte 1992; Witte & Allen 2000). According to these theories, the likelihood of an adaptive threat response (e.g., adhering to COVID-19 or climate change-related recommendations) increases with the extent to which a person perceives the situation as threatening (threat appraisal) and, subsequently, judges that they are able to cope with the threat (efficacy beliefs; Witte & Allen 2000).

Prior empirical work confirms the importance of threat appraisals for predicting responses to both public health issues and climate change (Harper et al. 2020; Hartmann et al. 2014; Jørgensen et al. 2021; Witte & Allen 2000). Here, we examine whether there are differences in the cognitive (perceived threat) and affective component (*fear*) of threat appraisal for COVID-19 and climate change. Within the cognitive component, we differentiate *personal threat*, *threat to close others*, and *threat to vulnerable others*. For global crises, like COVID-19 and climate change, both perceived threat to one’s own well-being (personal threat) and threat to others can motivate action (Corner et al. 2014; Ortega-Egea et al. 2014; Slater et al. 2015; Van der Linden et al. 2015). When it concerns others, a distinction can be made between close others who are important to people personally and vulnerable others; those who are especially vulnerable to a certain threat such as the elderly when it concerns COVID-19 (Christner et al. 2020).

Similarly, there is strong evidence for the importance of efficacy beliefs in motivating adaptive responses to threats (beliefs of being able to effectively respond to a threat, Bandura 1977; Chen 2015; Homburg & Stolberg 2006; Jørgensen et al. 2021; Witte & Allen 2000). Previous research highlights that large-scale global threats, which cannot be solved by an individual but require collective effort, may require beliefs about the efficacy of the collective in addition to personal efficacy beliefs (Chen 2015; Homburg & Stolberg 2006; Jugert et al. 2016). Here, we focus on *participative efficacy beliefs*, the belief that one can personally make an incremental difference in achieving the collective goal (Van Zomeren et al. 2013). Participative efficacy is an important predictor of collective action (Bamberg et al. 2015; Van Zomeren et al. 2013) and bridges the concepts of personal and collective efficacy, by taking into account the importance and indispensability of the individual’s actions towards achieving the collective goal. Prior work shows a lack of perceived efficacy in the context of climate change (Doherty & Webler 2016; Lorenzoni et al. 2007).

### Social norms

Both health and climate change behaviors are strongly influenced by beliefs about what others deem appropriate behavior (*injunctive social norms*) and about what others actually do (*descriptive social norms*, Bergquist et al. 2019; Cialdini et al. 1990; Mollen et al. 2013). Furthermore, given the collective nature of the COVID-19 pandemic and climate change crises, social norms might be especially important according to collective action theories (Fritsche et al. 2018; Reese et al. 2020).

### Perceptions of governmental policy

An effective, organized response to collective threats like COVID-19 and climate change also requires sensible government regulations and policies (Doherty & Webler 2016; Hart & Feldman 2016; Lubell 2002). In modern democracies, the success of governmental policies depends on public support and widespread motivation to act upon those policies. Governmental policy and recommendations have proven to make a great difference in combatting COVID-19 (Van Uffelen et al. 2020; Walker & Smith 2020). Similarly, governmental actions and policy have been shown to be related to climate change behaviors (Feldman & Hart 2016; Hart & Feldman 2016; Jamelske et al. 2013; Lubell 2002). Here, we therefore compare to what extent perceptions of *governmental policy* influence individual responses to COVID-19 and climate change.

### Current research

We investigate similarities and differences in the extent to which people *perceive* certain drivers as important for engaging in COVID-19 vs climate-friendly behaviors and in the *relationship* between these drivers and behaviors aiming to curb COVID-19 and climate change. Our aim is to inform climate-related interventions using insights about rapid and effective responses to the COVID-19 pandemic.

## Method

### Sample and design

We conducted an online survey among a snowball convenience sample recruited through the social media channels of a Dutch university (*N* = 536, 95% power to detect Cohen’s *d*_*z*_ = 0.14 and *f*^2^ = 0.04). Two participants showed no variance in all self-report responses, and were excluded from analyses, leaving a total of 534 participants (69.7% female, 27.9% male, 2.4% other/ “rather not say”; age ranged from 17 to 82, *M*_*age*_ = 38.10, *SD* = 14.03, *n* = 9 “rather not say”). The vast majority had a university degree (84.5%) and lived in Europe (94.8%, 68.7% in the Netherlands, *n* = 9 "rather not say"). The sample was thus highly educated in comparison to a more general sample.

### Procedure

Participants completed a survey hosted on Qualtrics which was available in Bulgarian, Dutch, English, German, and Icelandic. After providing informed consent, participants answered two blocks of identical questions rating the perceived importance of key drivers of behavior as well as the extent to which they themselves engage in COVID-19 and climate change-related behaviors. Block order was randomized. Lastly, participants had the opportunity to leave comments. All study procedures were approved by our university’s ethical review board (reference number: 2020-PC-12051).

### Measures

Each question block introduced the study topic as participants’ opinions, motivations, and behavior related to COVID-19 [climate change]. We further provided examples of each behavior category (e.g., COVID-19: social distancing, washing hands frequently; climate change: consuming less meat/dairy, lowering the thermostat) so that each participant could envision behaviors within each category that were most relevant to their personal life. Hereafter, we asked participants “To what extent do you engage in behavior to help in the fight against the coronavirus [climate change]?” (*Not at all* (1) to *A great deal* (5)), thus referring to behaviors in general rather than the examples mentioned. Because behavior was not normally distributed, we created a categorical variable with three categories for the COVID-19 and climate change behavior scales respectively, based on where on the scale significant clusters of participants were located.^1^ The three categories contain participants who reported relatively low, medium, and high frequencies of a given behavior. Next, participants were asked to indicate the importance of eight drivers of behaviors related to COVID-19 [climate change] (*Not at all important* (1) to *Extremely important* (5) – 1 item each) in randomized order: governmental policy, injunctive and descriptive social norms, participative efficacy, perceived threat (to me, to close others, and to vulnerable others), and fear (see Table 1 for items in the Appendix). Materials and data are available on OSF link: https://osf.io/bcuqh/.

## Results

### Importance of behavioral drivers

**Fig. 1:**
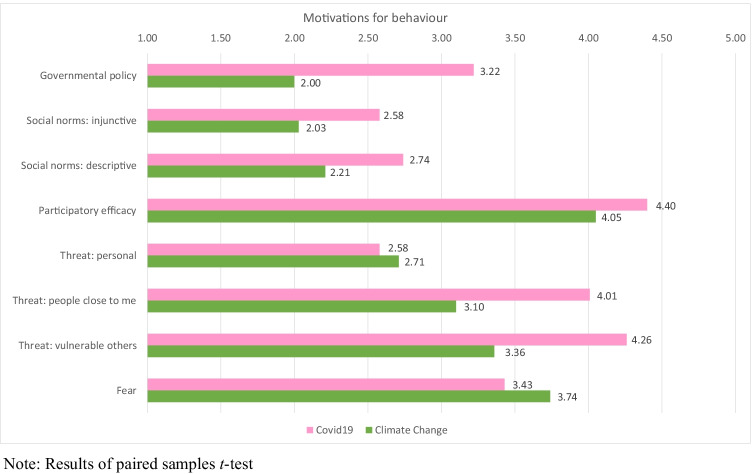
Average importance of theory-driven drivers for COVID-19 vs climate change. Note: Results of paired samples *t*-test

First, we examined the *perceived importance* of each behavioral driver in the context of COVID-19 and climate change (Fig. 1, Table 2 in the Appendix). Paired-sample *t* tests showed significant differences for the importance of all drivers when comparing COVID-19 and climate change. Most drivers were rated to be more important for acting to counter COVID-19. Only personal threat and fear of the potential impact of the crisis were rated to be more important for climate change, although effect sizes were small (*t*(533) =  − 2.00, *p* = 0.046, Cohen’s *d*_*z*_ = 0.09 and *t*(533) =  − 5.15, *p* < 0.001, Cohen’s *d*_*z*_ = 0.23 respectively). The greatest difference emerged for perceptions of governmental policy, which was rated to be more important for engaging in COVID-19 than climate change-related behaviors, *t*(533) = 27.76, *p* < 0.001, Cohen’s *d*_*z*_ = 1.20.^2^

### Relationship between behavioral drivers and self-reported behavior

Next, we investigated to what extent perceptions of the importance of behavioral drivers were *related* to actual, self-reported behavior. We conducted multinomial regressions, regressing behavior on each motive’s importance (Tables 3 and 4 in the Appendix). We investigated which drivers differentiated participants in the low and high compared to the medium frequency (reference) group, respectively controlling for gender, age, and education (for correlations between drivers and behaviors, see Tables 5 and 6 in the Appendix).

For COVID-19-related behaviors, participants who indicated the following drivers to be more important were more likely to report high (vs medium) frequency of behavior: participative efficacy (*B* = 0.53, SE = 0.17, Wald = 10.03, *p* = 0.002), threat to close others (*B* = 0.31, SE = 0.11, Wald = 7.51, *p* = 0.006), and governmental policy (*B* = 0.21, SE = 0.11, Wald = 3.98, *p* = 0.046). Participants, who indicated descriptive norms to be a more important driver (*B* =  − 0.49, SE = 0.17, Wald = 8.16, *p* = 0.004), were less likely to report low (vs medium) frequency behavior. In contrast to the literature, participants who indicated injunctive norms to be a less important driver (*B* = 0.33, SE = 0.16, Wald = 4.16, *p* = 0.041) were less likely to report low (vs medium) frequency behavior. In other words, participants who indicated that injunctive norms are an important driver reported a lower behavioral frequency.

For climate-friendly behaviors, participants who indicated that threat to vulnerable others (*B* = 0.37, SE = 0.11, Wald = 11.65, *p* = 0.001) and participative efficacy (*B* = 0.37, SE = 0.15, Wald = 5.90, *p* = 0.015) were important drivers and were more likely to report high (vs medium) frequency behavior. Participants who indicated that participative efficacy was a more important driver (*B* =  − 0.42, SE = 0.14, Wald = 9.60, *p* = 0.002) were less likely to report low (vs medium) frequency behavior.

## Discussion

In this study, we aimed to identify drivers that may help explain the stark difference in the behavioral response to the COVID-19 and climate change crises. Specifically, guided by behavioral change theories (Fishbein & Cappella 2006; Hornik & Woolf 1999), we aimed to identify (1) drivers that are important in instigating high-frequency behavior regarding the COVID-19 pandemic but not regarding climate change (candidates for *change*) and (2) drivers that are already important for behavior addressing both crises (candidates for *priming*). Results highlight governmental policy, threat to close others, and participative efficacy as key levers for further research and future interventions.

Government policy emerged as a candidate for *change* as it was perceived to be a more important behavioral driver in the COVID-19 than the climate change context. Furthermore, when investigating the relationship between drivers and self-reported behaviors, results showed that participants engaging in high (vs medium) frequency COVID-19 behaviors were more likely to indicate governmental policy as an important driver. This was not the case in the climate change context. Governments around the globe have acted quickly to address COVID-19. In contrast, governmental responses to climate change have been hesitant at best (e.g., although promising, the implementation of the Paris Agreement has remained insufficient). Interestingly, 22% of the participants who left a comment in the survey pointed out that the government should take a more leading role in the response to climate change. Consequently, if governments were seen as more supportive of climate-friendly policies and behaviors, this may stimulate individual climate-friendly behavior (Ockwell et al. 2009). Qualitative data from this study suggests that such policies would likely receive support from the population we sampled here, though future work should probe a broader population.

Additionally, threat to close others also emerged as a candidate for *change* as it was rated as a more important behavioral driver in the COVID-19 than the climate change context. Multinominal regressions investigating the relationships between drivers and self-reported behaviors furthermore showed that threat to close others was an important driver for high (vs medium) frequency behaviors related COVID-19, but not for climate change. Some participants also commented on the more pressing nature of COVID-19 versus climate change. People seemingly do not perceive climate change as threatening enough, which is in line with climate change being perceived as a rather distant threat (Ockwell et al. 2009; Weber 2006). This might also explain why personal threat and fear of the potential impact of the crisis were perceived as important drivers for climate-friendly behavior (more than those for COVID-19), but did not show a relationship with the engagement in behaviors that help limit climate change; the threat and fear might be too abstract and distant.

Furthermore, as a candidate for *priming*, we found that for both COVID-19 and climate change, participative efficacy beliefs were strongly related to high-frequency behavior (cf. Jørgensen et al. 2021, regarding COVID-19 behaviors). In line with the importance of participative efficacy for climate-friendly behavior, an Ipsos poll among > 28,000 adults (April, 2020) has shown that two-thirds of participants believed that the issue of climate change is as pressing as COVID-19, but also that people are not changing their behavior because they believe they are unable to make an impact (Gray & Jackson 2020). Priming participative efficacy beliefs for climate change by stressing the importance of everyone chipping in via environmental communication may thus accelerate climate-friendly behaviors.

This study provides a snapshot of key perceptions and action readiness at the height of the COVID-19 pandemic but is also limited in several ways. In order to capture perceptions at the right time during the developing COVID-19 pandemic, we recruited a cross-sectional convenience sample with limited generalizability. Replication of the current findings is desirable. For example, investigating whether and how drivers might differ for residents of developed vs developing countries (Thomas & Benjamin 2018), for different intrapersonal stages of behavior change (Prochaska et al. 2014), and among a sample that better reflects the general population as the current sample was, on average, more highly educated than the general population. Furthermore, to reduce participant burden, we used single item measures in line with common research practices (e.g., Boerman et al. 2012; Du et al. 2011; Swim & Geiger 2017). This raises potential concerns about reliability, but please note that research shows that single and multiple item measures perform similarly (Bergkvist & Rossiter 2007; Gardner et al. 1998). Furthermore, when measuring engagement in COVID-19 vs climate change behaviors, we allowed participants to focus on top-of-mind behaviors relevant to their own lives but provided guiding examples. Because drivers may vary between specific behaviors within a crisis category, follow-up research should distinguish between different behaviors.

Future research could also address the causal relationships between COVID-19 and climate change behaviors. While here we consider differences in the reasons behind engaging in behaviors that help in combatting climate change and COVID-19, prior work has highlighted that, under certain circumstances, the COVID-19 pandemic might positively influence pro-environmental actions (Tchetchik et al. 2021; but see also Ecker et al. 2020). A fruitful future avenue of work lies in the exploration of relationships between reasons motivating each behavior and subsequent effects on the respective other type of behavior. Lastly, it would be interesting to conduct a similar study when climate change is more prominently in the news than the COVID-19 pandemic. When the study was conducted, the seriousness of the COVID-19 pandemic only just sank in and was surrounded by great uncertainty. While there were also climate change-related natural disasters, for example, the aftermath of the Australian bushfires; floods in South America, Africa, and Asia; and bushfires in Siberia, COVID-19 was more prominent in the media in Europe. It would be interesting to investigate how this media prominence might influence COVID-19 and climate change-related behaviors (cf. Wonneberger et al. 2020) and how differential knowledge regarding governmental policies on COVID-19 vs climate change plays a role in this.

The current study exploratively investigated the drivers that play a role in behavior to combat the COVID-19 pandemic and climate change. We therefore recommend further exploration of our current findings (while keeping in mind the aforementioned limitations). Regarding practical implications, three strategies can be cautiously derived to stimulate climate-friendly behavior, although we commend these should first be tested in more detail. First, interventions focused on leveraging drivers that are related to COVID-19, but not to climate-friendly behavior. The results indicate that this can be done by *changing* perceptions regarding governmental policies and threat to close others. Changing perceptions regarding governmental policies could be done by developing environmental communication campaigns on changing the perception of governmental policies for the better. Additionally, this could be achieved by stimulating people to engage in public-sphere pro-environmental behavior (e.g., taking part in climate marches) to enhance the chance that environmental governmental policy will change. Threat to close others could be made more salient by, for example, using virtual reality experiences showing how climate change can also affect people’s own environment, showing that also their close others are susceptible to the threat of climate change. Second, interventions focusing on already important drivers by *priming* these motives, like participative efficacy, could be highly effective. Therefore, communicating to people that their individual actions are indispensable to reach the goal of combating climate change could be an effective way of stimulating climate-friendly behavior.

## Data Availability

The data that support the findings of this study are openly available on the Open Science Framework via this link https://osf.io/bcuqh/.

## Appendix

**Table 1:** Items to measure drivers for engaging in behaviors that help fight COVID-19 and climate change

Governmental policy	Because the government is advocating for these behaviors
Injunctive social norms *(Árnadóttir, Kok, Van Gils, & Ten Hoor,* 2019)	Because people who are important to me think I should engage in these behaviors
Descriptive social norms *(**Árnadóttir* *et* *al*. 2019*)*	Because most people who are important to me are engaging in these behaviors
Participative efficacy *(**Van Zomeren et al.* 2013*)*	Because if I engage in these behaviors, we can together limit [the spread of the coronavirus/the harm caused by climate change]
Perceived threat to me *(**Slater et al.* 2015*)*	Because [the coronavirus/climate change] poses a serious threat to me personally
Perceived threat to those close others *(**Slater et al.* 2015*)*	Because [the coronavirus/climate change] poses a serious threat to people who are important to me
Perceived threat to vulnerable others *(**Slater et al.* 2015*)*	Because [the coronavirus/climate change] poses a serious threat to vulnerable others in society
Fear *(**Hartmann et al.* 2014*)*	Because I am scared of the potential impact of [the coronavirus/climate change]

*Note*. The references indicate research the item was based on.

**Table 2:** Means and standard deviations for the drivers for engaging in behaviors that help fight COVID-19 and climate change, and results of paired samples *t*-test comparing the drivers for the two crises

	COVID-19		Climate change		***t***	***p***	***Cohen’s d***_***z***_
	Mean	SD	Mean	SD			
Governmental policy	3.22	(1.03)	2.00	(0.90)	27.76	< .001	1.20
Social norms – injunctive	2.58	(1.13)	2.03	(0.96)	11.11	< .001	0.48
Social norms – descriptive	2.74	(1.12)	2.21	(0.94)	11.13	< .001	0.48
Participative efficacy	4.40	(0.78)	4.05	(0.97)	7.80	< .001	0.34
Personal threat	2.58	(1.23)	2.71	(1.17)	− 2.00	.046	0.09
Threat to close others	4.01	(1.10)	3.10	(1.22)	14.47	< .001	0.63
Threat to vulnerable others	4.26	(0.88)	3.36	(1.21)	17.14	< .001	0.74
Fear	3.43	(1.17)	3.74	(1.10)	− 5.15	< .001	0.23

**Table 3:** Results of multinomial regressions from self-reported behaviors that help fight COVID-19 on the behavioral drivers

	*b*	SE	Wald	*p*	Odds ratio	95% CI for odds ratio	
						Lower	Upper
Low vs. medium							
Intercept	− 0.08	1.03	0.01	.934			
Governmental policy	0.05	0.14	0.14	.705	1.06	0.80	1.40
Social norms – injunctive	0.33	0.16	4.16	.041	1.40	1.01	1.92
Social norms – descriptive	− 0.49	0.17	8.16	.004	0.61	0.44	0.86
Participative efficacy	0.02	0.19	0.01	.919	1.02	0.70	1.49
Personal threat	− 0.12	0.15	0.70	.404	0.88	0.66	1.18
Threat to close ones	0.20	0.14	2.07	.150	1.22	0.93	1.60
Threat to vulnerable others	− 0.04	0.18	0.04	.840	0.97	0.68	1.36
Fear	− 0.12	0.15	0.65	.421	0.89	0.67	1.19
Gender (female vs. rest)	0.18	0.30	0.35	.555	1.19	0.66	2.14
Age	− 0.02	0.01	2.73	.099	0.98	0.96	1.00
Education level: university vs. rest	− 0.30	0.36	0.71	.399	0.74	0.36	1.50
High vs. medium							
Intercept	− 4.39	0.92	22.58	< .001			
Governmental policy	0.21	0.11	3.98	.046	1.24	1.00	1.52
Social norms – injunctive	− 0.13	0.12	1.13	.288	0.88	0.69	1.12
Social norms – descriptive	− 0.24	0.12	3.66	.056	0.79	0.62	1.01
Participative efficacy	0.53	0.17	10.02	.002	1.70	1.22	2.36
Personal threat	0.15	0.10	2.22	.136	1.16	0.95	1.42
Threat to close ones	0.31	0.11	7.51	.006	1.36	1.09	1.70
Threat to vulnerable others	0.06	0.14	0.18	.671	1.06	0.81	1.39
Fear	0.02	0.11	0.05	.822	1.03	0.83	1.27
Gender (female vs. rest)	0.23	0.22	1.08	.299	1.26	0.81	1.96
Age	0.00	0.01	0.21	.651	1.00	0.99	1.02
Education level: university vs. rest	− 0.05	0.29	0.04	.851	0.95	0.54	1.66

“Medium” is the specified reference category. For comparing low vs medium frequency behaviors, this means that minus values should be interpreted as drivers being more important in the medium compared to the low category, whereas for comparing medium vs high frequency behaviors, this means that minus values should be interpreted as drivers being more important in the medium compared to the high category (*n* = 525).

*R*^*2*^ = 0.14 (Cox and Snell), *R*^2^ = 0.16 (Nagelkerke). Model *χ*^2^ (22) = 78.33, *p* < 0.001

**Table 4:** Results of multinomial regressions from self-reported behaviors that help fight climate change on the behavioral drivers

	b	SE	Wald	*p*	Odds ratio	95% CI for odds ratio	
						Lower	Upper
Low vs. medium							
Intercept	2.65	0.74	12.69	< .001			
Governmental policy	0.12	0.14	0.77	.379	1.13	0.86	1.49
Social norms – injunctive	− 0.13	0.17	0.56	.454	0.88	0.63	1.23
Social norms – descriptive	0.02	0.17	0.01	.929	1.02	0.73	1.42
Participative efficacy	− 0.42	0.13	9.60	.002	0.66	0.51	0.86
Personal threat	− 0.08	0.12	0.45	.501	0.92	0.72	1.17
Threat to close ones	− 0.04	0.12	0.15	.698	0.96	0.76	1.20
Threat to vulnerable others	− 0.18	0.11	2.57	.109	0.84	0.67	1.04
Fear	− 0.21	0.13	2.78	.096	0.81	0.63	1.04
Gender (female vs. rest)	0.12	0.25	0.21	.649	1.12	0.68	1.85
Age	− 0.01	0.01	2.29	.130	0.99	0.97	1.00
Education level (university vs. rest)	0.68	0.35	3.92	.048	1.98	1.01	3.90
High vs. medium							
Intercept	− 2.49	0.84	8.88	.003			
Governmental policy	− 0.03	0.13	0.04	.844	0.97	0.75	1.26
Social norms – injunctive	0.00	0.16	0.00	.979	1.00	0.74	1.36
Social norms – descriptive	− 0.28	0.16	3.14	.076	0.75	0.55	1.03
Participative efficacy	0.37	0.15	5.90	.015	1.45	1.07	1.96
Personal threat	− 0.08	0.11	0.51	.474	0.92	0.74	1.15
Threat to close ones	0.02	0.11	0.05	.824	1.02	0.83	1.27
Threat to vulnerable others	0.37	0.11	11.65	.001	1.45	1.17	1.80
Fear	0.16	0.13	1.70	.192	1.18	0.92	1.51
Gender (female vs. rest)	0.09	0.25	0.14	.710	1.10	0.67	1.80
Age	− 0.02	0.01	3.45	.063	0.99	0.97	1.00
Education level (university vs. rest)	− 0.09	0.29	0.09	.769	0.92	0.52	1.63

“Medium” is the specified reference category. For comparing low vs medium frequency behaviors, this means that minus values should be interpreted as drivers being more important in the medium compared to the low category, whereas for comparing medium vs high frequency behaviors, this means that minus values should be interpreted as drivers being more important in the medium compared to the high category (*n* = 525).

*R*^*2*^= 0.22 (Cox and Snell), *R*^2^ = 0.25 (Nagelkerke). Model *χ*^2^ (22) = 132.58, *p* < 0.001

**Table 5:** Correlations between COVID-19 behavior and the perceived importance of behavioral drivers

	Behavior	Governmental policy	Social norms – injunctive	Social norms – descriptive	Participative efficacy	Personal threat	Threat to close ones	Threat to vulnerable others	Fear
Behavior	–	0.100 (0.021)	− 0.073 (0.093)	0.006 (0.883)	0.226 (< 0.001)	0.177 (< 0.001)	0.177 (< 0.001)	0.146 (0.001)	0.186 (< 0.001)
Governmental policy	0.077 (0.088)	–	0.253 (< 0.001)	0.301 (< 0.001)	0.185 (< 0.001)	0.126 (0.003)	0.020 (0.651)	0.187 (< 0.001)	0.112 (0.009)
Social norms – injunctive	− 0.106 (0.020)	0.256 (< 0.001)	–	0.667 (< 0.001)	-0.004 (0.927)	0.185 (< 0.001)	0.124 (0.004)	0.063 (0.149)	0.150 (0.001)
Social norms – descriptive	-0.032 (0.482)	0.286 (< 0.001)	0.653 (< 0.001)	–	0.069 (0.112)	0.192 (< 0.001)	0.157 (< 0.001)	0.119 (0.006)	0.226 (< 0.001)
Participative efficacy	0.190 (< 0.001)	0.176 (< 0.001)	0.011 (0.815)	0.063 (0.167)	–	0.128 (0.003)	0.275 (< 0.001)	0.461 (< 0.001)	0.349 (< 0.001)
Personal threat	0.130 (0.004)	0.115 (0.011)	0.170 (< 0.001)	0.162 (< 0.001)	0.129 (0.004)	–	0.396 (< 0.001)	0.109 (0.012)	0.512 (< 0.001)
Threat to close ones	0.134 (0.003)	0.010 (0.833)	0.108 (0.017)	0.120 (0.008)	0.239 (< 0.001)	0.361 (< 0.001)	–	0.362 (< 0.001)	0.422 (< 0.001)
Threat to vulnerable others	0.126 (0.005)	0.177 (< 0.001)	0.049 (0.281)	0.112 (0.013)	0.429 (< 0.001)	0.110 (0.015)	0.346 (< 0.001)	–	0.273 (< 0.001)
Fear	0.145 (0.001)	0.067 (0.140)	0.153 (0.001)	0.205 (< 0.001)	0.333 (< 0.001)	0.475 (< 0.001)	0.394 (< 0.001)	0.272 (< 0.001)	–

The upper right half of the table represents the bivariate correlations (*n* = 534), the lower left half represents the partial correlations (*df* = 484) — controlled for demographics (gender, age, education, country of residence) and political affiliation. All items were measured on a 1–5 scale. The first number represents the correlation; between brackets, the exact *p*-values are reported.

**Table 6:** Correlations between climate-friendly behavior and the perceived importance of behavioral drivers

	Behavior	Governmental policy	Social norms – injunctive	Social norms – descriptive	Participative efficacy	Personal threat	Threat to close ones	Threat to vulnerable others	Fear
Behavior	–	-0.013 (0.758)	0.016 (0.704)	-0.040 (0.352)	0.388 (< 0.001)	0.173 (< 0.001)	0.219 (< 0.001)	0.363 (< 0.001)	0.309 (< 0.001)
Governmental policy	− 0.020 (0.655)	–	0.340 (< 0.001)	0.362 (< 0.001)	0.065 (0.136)	0.194 (< 0.001)	0.199 (< 0.001)	0.102 (0.018)	0.159 (< 0.001)
Social norms – injunctive	− 0.004 (0.937)	0.323 (< 0.001)	–	0.674 (< 0.001)	0.059 (0.171)	0.247 (< 0.001)	0.175 (< 0.001)	0.138 (0.001)	0.240 (< 0.001)
Social norms – descriptive	− 0.068 (0.137)	0.352 (< 0.001)	0.664 (< 0.001)	–	0.065 (0.135)	0.222 (< 0.001)	0.183 (< 0.001)	0.119 (0.006)	0.223 (< 0.001)
Participative efficacy	0.339 (< 0.001)	0.077 (0.091)	0.046 (0.309)	0.052 (0.249)	–	0.228 (< 0.001)	0.310 (< 0.001)	0.380 (< 0.001)	0.458 (< 0.001)
Personal threat	0.131 (0.004)	0.178 (< 0.001)	0.214 (< 0.001)	0.189 (< 0.001)	0.195 (< 0.001)	–	0.451 (< 0.001)	0.341 (< 0.001)	0.436 (< 0.001)
Threat to close ones	0.174 (< 0.001)	0.214 (< 0.001)	0.173 (< 0.001)	0.160 (< 0.001)	0.277 (< 0.001)	0.441 (< 0.001)	–	0.432 (< 0.001)	0.319 (< 0.001)
Threat to vulnerable others	0.289 (<0.001)	0.122 (0.007)	0.131 (0.004)	0.102 (0.024)	0.314 (< 0.001)	0.322 (< 0.001)	0.394 (< 0.001)	–	0.348 (< 0.001)
Fear	0.234 (< 0.001)	0.165 (< 0.001)	0.246 (< 0.001)	0.236 (< 0.001)	0.417 (< 0.001)	0.428 (< 0.001)	0.300 (< 0.001)	0.286 (< 0.001)	–

The upper right half of the table represents the bivariate correlations (*n* = 534); the lower left half represents the partial correlations (*df* = 484) — controlled for demographics (gender, age, education, country of residence) and political affiliation. All items were measured on a 1–5 scale. The first number represents the correlation; between brackets, the exact *p*-values are reported.

## Footnotes

1 For COVID-19-related behaviors, participants who indicated to engage “not at all,” “slightly,” or “moderately” in COVID-19-related behavior were grouped in the “low” frequency group (14.0%). Participants who indicated to engage “considerably” in COVID-19-related behavior were grouped in the “medium” frequency group (46.1%), whereas participants who indicated to engage “a great deal” in COVID-19-related behavior were grouped in the “high” frequency group (39.9%). For climate change-related behaviors, participants who indicated to engage “not at all” or “slightly” in climate change-related behavior were grouped in the “low” frequency group (26.8%). Participants who indicated to engage “moderately” in climate change-related behavior were grouped in the “medium” frequency group (42.3%), whereas participants who indicated to engage “considerably” or “a great deal” in climate change-related behavior were grouped in the “high” frequency group (30.9%).

2 Checking for order effects (whether the questions pertaining climate change or COVID-19 were asked first) revealed no meaningful differences.

## Code availability

NA.

## References

[CR1] Árnadóttir ÁD, Kok G, Van Gils S, Ten Hoor GA (2019). Waste separation in cafeterias: a study among university students in the netherlands. Int J Environ Res Public Health.

[CR2] Bamberg S, Rees J, Seebauer S (2015). Collective climate action: determinants of participation intention in community-based pro-environmental initiatives. J Environ Psychol.

[CR3] Bandura A (1977). Self-efficacy: toward a unifying theory of behavioral change. Psychol Rev.

[CR4] Bergkvist L, Rossiter JR (2007). The predictive validity of multiple-item versus single-item measures of the same constructs. J Mark Res.

[CR5] Bergquist M, Nilsson A, Schultz WP (2019). A meta-analysis of field-experiments using social norms to promote pro-environmental behaviors. Glob Environ Change.

[CR6] Boerman SC, Reijmersdal EA, Neijens PC (2012). Sponsorship disclosure: effects of duration on persuasion knowledge and brand responses. J Commun.

[CR7] Chen M (2015). Self-efficacy or collective efficacy within the cognitive theory of stress model: which more effectively explains people's self-reported proenvironmental behavior?. J Environ Psychol.

[CR8] Christner N, Sticker RM, Söldner L, Mammen M, Paulus M (2020) Prevention for oneself or others? Psychological and social factors that explain social distancing during the COVID-19 pandemic. J Health Psychol 10.1177/1359105320980793 PMC9036152 33302730

[CR9] Cialdini RB, Reno RR, Kallgren CA (1990). A focus theory of normative conduct: recycling the concept of norms to reduce littering in public places. J Pers Soc Psychol.

[CR10] Cooper DH, Nagel J (2021) Lessons from the pandemic: climate change and COVID-19. International Journal of Sociology and Social Policy

[CR11] Corner A, Markowitz E, Pidgeon N (2014). Public engagement with climate change: the role of human values. Wiley Interdiscip Rev Clim Change.

[CR12] Cova F (2020) Individual differences and reactions to the coronavirus outbreak a virus_Study1_Report. corona virus study report. Retrieved from https://docs.google.com/document/d/1hb6nYtETS8TirPSgJ01BQsteDFj15s78SCrj29vnSnk/edit?usp=embed_facebook. Retrieved March 2020

[CR13] Doherty KL, Webler TN (2016). Social norms and efficacy beliefs drive the alarmed segment’s public-sphere climate actions. Nat Clim Chang.

[CR14] Du S, Bhattacharya CB, Sen S (2011). Corporate social responsibility and competitive advantage: overcoming the trust barrier. Manage Sci.

[CR15] Ecker UK, Butler LH, Cook J, Hurlstone MJ, Kurz T, Lewandowsky S (2020). Using the COVID-19 economic crisis to frame climate change as a secondary issue reduces mitigation support. J Environ Psychol.

[CR16] Feldman L, Hart PS (2016). Using political efficacy messages to increase climate activism: the mediating role of emotions. Sci Commun.

[CR17] Fishbein M, Cappella JN (2006). The role of theory in developing effective health communications. J Commun.

[CR18] Fritsche I, Barth M, Jugert P, Masson T, Reese G (2018). A social identity model of pro-environmental action (SIMPEA). Psychol Rev.

[CR19] Galbraith E, Otto R (2020) Coronavirus response proves the world can act on climate change. the conversation. Retrieved from http://theconversation.com/coronavirus-response-proves-the-world-can-act-on-climate-change-133999. Retrieved May 2020

[CR20] Gardner DG, Cummings LL, Dunham RB, Pierce JL (1998). Single-item versus multiple-item measurement scales: an empirical comparison. Educ Psychol Measur.

[CR21] Gray E, Jackson C (2020) Two thirds of citizens around the world agree climate change is as serious a crisis as coronavirus. Retrieved from https://www.ipsos.com/en/two-thirds-citizens-around-world-agree-climate-change-serious-crisis-coronavirus. Retrieved in May 2020

[CR22] Harper CA, Satchell LP, Fido D, Latzman RD (2020) Functional fear predicts public health compliance in the COVID-19 pandemic. International Journal of Mental Health and Addiction 10.1007/s11469-020-00281-5 PMC7185265 32346359

[CR23] Hart PS, Feldman L (2016). The influence of climate change efficacy messages and efficacy beliefs on intended political participation. PloS One.

[CR24] Hartmann P, Apaolaza V, D'Souza C, Barrutia JM, Echebarria C (2014) Environmental threat appeals in green advertising: the role of fear arousal and coping efficacy. Int J Advert: The Quarterly Review of Marketing Communications 33(4), Sefe.

[CR25] Homburg A, Stolberg A (2006). Explaining pro-environmental behavior with a cognitive theory of stress. J Environ Psychol.

[CR26] Hornik R, Woolf KD (1999). Using cross-sectional surveys to plan message strategies. Soc Mark Q.

[CR27] Jagers SC, Harring N, Löfgren Å, Sjöstedt M, Alpizar F, Brülde B, Dupont S (2020). On the preconditions for large-scale collective action. Ambio.

[CR28] Jamelske E, Barrett J, Boulter J (2013). Comparing climate change awareness, perceptions, and beliefs of college students in the united states and china. J Environ Stud Sci.

[CR29] Johns Hopkins University & Medicine (2020) Johns Hopkins Coronavirus Resource Center. Retrieved from https://coronavirus.jhu.edu/. Retrieved in June 2020

[CR30] Jørgensen F, Bor A, Petersen MB (2021). Compliance without fear: individual-level protective behaviour during the first wave of the COVID-19 pandemic. Br J Health Psychol.

[CR31] Jugert P, Greenaway KH, Barth M, Büchner R, Eisentraut S, Fritsche I (2016). Collective efficacy increases pro-environmental intentions through increasing self-efficacy. J Environ Psychol.

[CR32] Lorenzoni I, Nicholson-Cole S, Whitmarsh L (2007). Barriers perceived to engaging with climate change among the UK public and their policy implications. Global Environmental Change-Human and Policy Dimensions.

[CR33] Lubell M (2002). Environmental activism as collective action. Environ Behav.

[CR34] Michie S, Van Stralen MM, West R (2011). The behaviour change wheel: a new method for characterising and designing behaviour change interventions. Implement Sci.

[CR35] Mollen S, Rimal RN, Ruiter RAC, Kok G (2013). Healthy and unhealthy social norms and food selection. findings from a field-experiment. Appetite.

[CR36] Munck af Rosenschöld J, Rozema JG, Frye-Levine LA (2014) Institutional inertia and climate change: a review of the new institutionalist literature. Wiley Interdiscip Rev Clim Change 5(5):639–648

[CR37] Ockwell D, Whitmarsh L, O'Neill S (2009). Reorienting climate change communication for effective mitigation: forcing people to be green or fostering grass-roots engagement?. Sci Commun.

[CR38] Ortega-Egea JM, Garcia-de-Frutos N, Antolin-Lopez R (2014). Why do some people do “more” to mitigate climate change than others? Exploring heterogeneity in psycho-social associations. PloS One.

[CR39] Prochaska JJ, Fromont SC, Delucchi K, Young-Wolff KC, Benowitz NL, Hall S, Hall SM (2014) Multiple risk-behavior profiles of smokers with serious mental illness and motivation for change. Health Psychol 33(12):1518 10.1037/a0035164 PMC4425305 24467257

[CR40] Rainear AM, Christensen JL (2017). Protection motivation theory as an explanatory framework for proenvironmental behavioral intentions. Commun Res Rep.

[CR41] Reese G, Hamann K, Heidbreder LM, Loy L, Menzel C, Neubert S, Wullenkord MC (2020) SARS-CoV-2 and environmental protection: a collective psychology agenda for environmental psychology research. J Environ Psychol 70:101444 10.1016/j.jenvp.2020.101444 PMC7267801 32528209

[CR42] Rogers RW (1975). A protection motivation theory of fear appeals and attitude change1. J Psychol.

[CR43] Schmidt RC (2021) Are there similarities between the corona and the climate crisis? J Environ Stud Sci, 1–5 10.1007/s13412-021-00666-5 PMC7845791 33552836

[CR44] Segalov M (2020) The parallels between coronavirus and climate crisis are obvious. Retrieved from https://www.theguardian.com/environment/2020/may/04/parallels-climate-coronavirus-obvious-emily-atkin-pandemic. Retrieved September 2021

[CR45] Slater MD, Hayes AF, Chung AH (2015). Injury news coverage, relative concern, and support for alcohol-control policies: an impersonal impact explanation. J Health Commun.

[CR46] Swim JK, Geiger N (2017). From alarmed to dismissive of climate change: a single item assessment of individual differences in concern and issue involvement. Environ Commun.

[CR47] Tchetchik A, Kaplan S, Blass V (2021). Recycling and consumption reduction following the COVID-19 lockdown: the effect of threat and coping appraisal, past behavior and information. Resour Conserv Recycl.

[CR48] Thomas A, Benjamin L (2018). Perceptions of climate change risk in the bahamas. J Environ Stud Sci.

[CR49] Urbanovich T, Bevan JL (2020) Promoting environmental behaviors: applying the health belief model to diet change. Environ Commun 14(5):657–671

[CR50] Van Bavel JJ, Baicker K, Boggio PS, Capraro V, Cichocka A, Cikara M, Druckman JN (2020) Using social and behavioural science to support COVID-19 pandemic response. Nat Hum Behav 4:460–471 10.1038/s41562-020-0884-z 32355299

[CR51] Van der Linden S, Maibach E, Leiserowitz A (2015). Improving public engagement with climate change: five “best practice” insights from psychological science. Perspect Psychol Sci.

[CR52] Van Uffelen X, Frijters S, Arjovic S (2020) De belangrijkste grafieken en kaarten over de uitbraak van het coronavirus in nederland. *De Volkskrant* Retrieved from https://www.volkskrant.nl/nieuws-achtergrond/de-belangrijkste-grafieken-en-kaarten-over-de-uitbraak-van-het-coronavirus-in-Nederland~b18f4613/. Retrieved June 2020

[CR53] van Valkengoed AM, Steg L (2019). Meta-analyses of factors motivating climate change adaptation behaviour. Nat Clim Chang.

[CR54] Van Zomeren M, Postmes T, Spears R (2008). Toward an integrative social identity model of collective action: a quantitative research synthesis of three socio-psychological perspectives. Psychol Bull.

[CR55] Van Zomeren M, Saguy T, Schellhaas FM (2013). Believing in “making a difference” to collective efforts: participative efficacy beliefs as a unique predictor of collective action. Group Process Intergroup Relat.

[CR56] Walker S, Smith H (2020) Why has Eastern Europe suffered less from coronavirus than the west? *The Guardian*. Retrieved from https://www.theguardian.com/world/2020/may/05/why-has-eastern-europe-suffered-less-from-coronavirus-than-the-west. Retrieved June 2020

[CR57] Weber EU (2006). Experience-based and description-based perceptions of long-term risk: why global warming does not scare us (yet). Clim Change.

[CR58] Whitmarsh L, O’Neill S, Lorenzoni I (2013). Public engagement with climate change: what do we know and where do we go from here?. Int J Media Cult Politics.

[CR59] Witte K (1992). Putting the fear back into fear appeals: the extended parallel process model. Communications Monographs.

[CR60] Witte K, Allen M (2000). A meta-analysis of fear appeals: implications for effective public health campaigns. Health Educ Behav.

[CR61] Wonneberger A, Meijers MHC, Schuck ART (2020). Shifting public engagement: how media coverage of climate change conferences affects climate change audience segments. Public Underst Sci.

