# Correction to: Learning from the COVID-19 pandemic to combat climate change: comparing drivers of individual action in global crises

**DOI:** 10.1007/s13412-022-00749-x

**Published:** 2022-01-19

**Authors:** Marijn H. C. Meijers, Christin Scholz, Ragnheiður “Heather” Torfadóttir, Anke Wonneberger, Marko Markov

**Affiliations:** 1 Amsterdam School of Communication Research, Department of Communication Science, University of Amsterdam, PO BOX 15791, 1001 NG Amsterdam, The Netherlands; 2 Department of Communication Science, University of Amsterdam, PO BOX 15791, 1001 NG Amsterdam, The Netherlands

**Correction to: Journal of Environmental Studies and Sciences**

https://doi.org/10.1007/s13412-021-00727-9

The original version of this article unfortunately contained a mistake as a dot was omitted. The data in Table 5, 2^nd^ row under the header Behavior should be 0.077 (0.088) instead of 0077 (0.088).

The original article has been corrected.

